# Lipid Mediators Are Critical in Resolving Inflammation: A Review of the Emerging Roles of Eicosanoids in Diabetes Mellitus

**DOI:** 10.1155/2015/568408

**Published:** 2015-03-19

**Authors:** Fernando H. G. Tessaro, Thais S. Ayala, Joilson O. Martins

**Affiliations:** Laboratory of Immunoendocrinology, Department of Clinical and Toxicological Analyses, Faculty of Pharmaceutical Sciences, University of São Paulo, Avenida Professor Lineu Prestes 580, Bloco 17, 05508-000 São Paulo, SP, Brazil

## Abstract

The biosynthesis pathway of eicosanoids derived from arachidonic acid, such as prostaglandins and leukotrienes, relates to the pathophysiology of diabetes mellitus (DM). A better understanding of how lipid mediators modulate the inflammatory process may help recognize key factors underlying the progression of diabetes complications. Our review presents recent knowledge about eicosanoid synthesis and signaling in DM-related complications, and discusses eicosanoid-related target therapeutics.

## 1. Introduction

**Figure 1:**
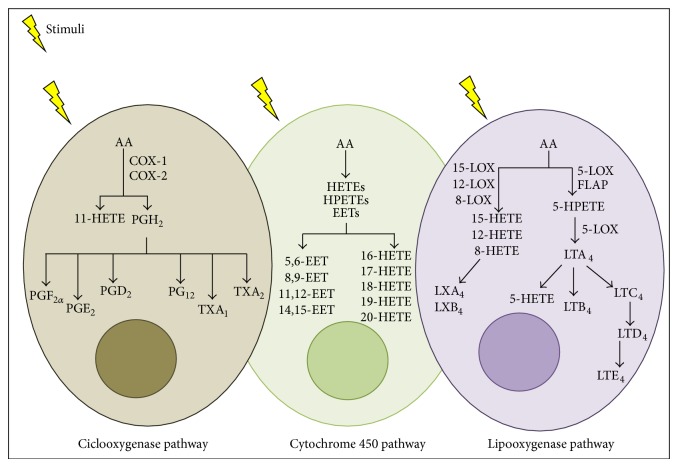
Eicosanoid synthesis pathways. After cell stimulation, arachidonic acid (AA) can be metabolized by three enzymes: cyclooxygenase (COX), lipoxygenase (LOX), and cytochrome P450 (CYP 450). COX catalyzes AA in (prostaglandin) PGG_2_ and PGH_2_, and these are converted into PGD_2_, PGE_2_, PGF_2*α*_, PG_12_, TXA_1_, and TXA_2_. The LOX pathway catalyzes AA into hydroxyeicosatetraenoic acids (HETEs) and diverse hydroperoxyeicosatetraenoic acids (HPETEs). This pathway involves four enzymes: 5-LOX, 8-LOX, 12-LOX, and 15-LOX. 5-LOX interacts with a 5-LOX-activating protein (FLAP), enhancing the interaction of 5-LOX to AA. LTA_4_ hydrolases convert LTA_4_ into LTB_4_, and LTC_4_ synthase can convert LTA_4_ to LTC_4_, whereupon it is then metabolized to LTD_4_ and LTE_4_. 5-LOX synthetizes LXA_4_ and LXB_4_ using 15-HETE. The pathway of CYP-450 leads to the conversion of HETEs, including 16-, 17-, 18-, 19-, and 20-HETE and epoxyeicosatrienoic acids (EETs): 5,6-, 8,9-, 11,12-, and 14,15-EET.

**Figure 2:**
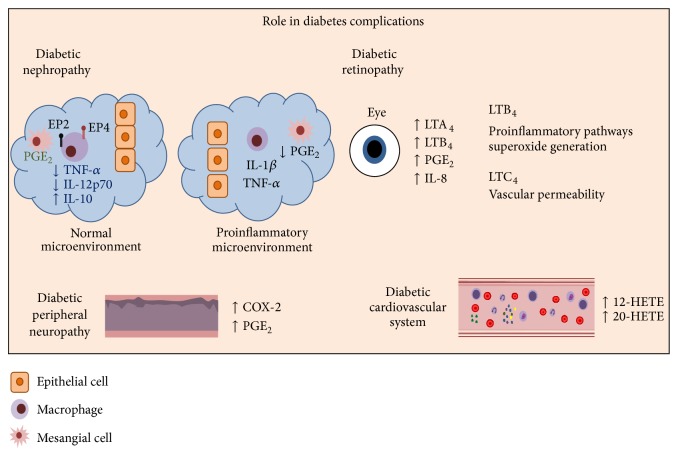
Eicosanoid compounds affect different organs in diabetes complications. Diabetic nephropathy, one of the most common complications in diabetes, shows low PGE_2_ levels and altered glomerular hemodynamics. This dilates arteries and increases microvascular permeability. In normal conditions PGE_2_ downregulates TNF-*α* production and upregulates IL-10 production through EP2 and EP4 receptor signaling. However, a proinflammatory environment leads to cell permeabilization, low concentrations of PGE_2_, and mesangial cell proliferation. Diabetic retinopathy is another common complication in diabetes. In diabetes, the environment in the retina has a particular lipid profile, with higher COX-2 and abnormal production of PG. LTA_4_ and LTB_4_ are enhanced in addition to IL-8. Diabetic peripheral neuropathy is correlated with high COX-2 and PGE_2_. In a diabetic's cardiovascular system, PGE_2_ has an important role in microvascular permeability, and 12-HETE and 20-HETE lower the activity of endothelial progenitor cell (EPC) function.

Eicosanoids are biologically active lipid mediators that regulate inflammation [1] and that include prostaglandins (PGs), prostacyclins, thromboxanes (TX), leukotrienes (LT), and lipoxins (LX) (Figure 1) [2–4]. They may amplify or reduce inflammation, which coordinates cytokine production, antibody formation, cell proliferation and migration, and antigen presentation [2, 5, 6]. To prevent great tissue damage, eicosanoids also control the inflammatory resolution and tissue repair process [7, 8]. Imbalances in eicosanoid synthesis have been reported to drive chronic inflammation [1, 9], which deregulates signaling pathways and/or cellular events leading to abnormal immune functions [6, 10]. In particular, circulating and local mediators, such as eicosanoids, interleukin- (IL-) 1*β*, tumor necrosis factor- (TNF-) *α*, IL-6, IL-8, macrophage migration inhibitory factor (MIF), and free radicals, create a state of low-chronic inflammation in diabetic patients [5, 10, 11]. Inflammation may lead to diabetes progression, including damage to the kidneys (diabetic nephropathy), eyes (diabetic retinopathy), nerves (diabetic neuropathy), and cardiovascular system [12] (Figure 2).

In this review, we summarize the role of eicosanoids on the pathogenesis and progression of diabetes. In addition, we review drugs used to treat diabetic complications by acting on compounds of the eicosanoid pathway and speculate on possible future targets to treat diabetes complications.

## 2. The Role of Eicosanoids in Diabetes

The level of inflammation severity in diabetes is associated with hemoglobin A1 levels [13]. Increased PGE_2_ levels are related to dysfunction in insulin-regulated glycogen synthesis and gluconeogenesis in the liver [14, 15]. 12- as well as 15-hydroxyeicosatetraenoic acid (HETE) increases inflammatory cytokine expression, such as IL-6, TNF-*α*, and MCP-1, inducing chronic inflammation and the infiltration of inflammatory cells in adipose tissue [16–18]. In addition, 12-lipoxygenase (LOX) metabolites impair insulin action in adipocytes and can downregulate glucose transport, both of which may lead to insulin resistance [18, 19]. Nimesulide and metformin improved acute inflammation and impaired glucose metabolism [20], suggesting that impairing functions of prostaglandin synthesis are mediated by altered glucose levels [21].

### 2.1. Diabetic Nephropathy

Diabetic nephropathy is the major cause of diabetes-related death [22]. Renal disorders associated with diabetic nephropathy consist of modifications in renal hemodynamics, glomerular hypertrophy, mesangial cell proliferation, matrix accumulation, and proteinuria [23]. In normal conditions, PGE_2_ is the major PG in the kidneys and acts in renal physiology, glomerular filtration, and renin release [24, 25]. PGE_2_ activates kidney EP receptors, such as EP1, EP2, EP3, and EP4 in the collecting duct (except for EP2 whose mRNA has been localized to the outer and inner medulla of the kidney and EP4 which can also be expressed in the glomerulus) [25, 26]. Interactions between resident renal cells and macrophages change the microenvironment to a proinflammatory state, contributing to tissue damage and scarring [27, 28]. Macrophages and T cells infiltrate the glomeruli and interstitium, contributing to chronic renal failure in diabetic patients [27, 29–31].

During inflammation, macrophages release IL-1B and TNF-*α*, inducing endothelial cell permeability, altering glomerular hemodynamics, and decreasing PGE_2_ production by mesangial cells [32]. Normal levels of PGE_2_ suppress Th1 immune responses [33] and downregulate TNF-*α* production and upregulate IL-10 production through EP2 and EP4 receptor signaling, ending nonspecific inflammation [33–35]. Through an IL-10-dependent mechanism, PGE_2_ regulates IL-12 secretion by selectively inhibiting IL-12p70 production and stimulating IL-12p40 release [36, 37]. However, PGE_2_ is reduced in diabetic nephropathy, and this plays an essential role in the evolution of diabetic renal injury, strengthening the conclusion that inflammatory mechanisms have a significant role in both diabetic nephropathy development and progression [38–40]. Knockout podocyte-specific mice are protected against diabetes-induced nephropathy and albuminuria, showing the importance of COX-2 metabolites in the establishment of diabetic nephropathy [41].

### 2.2. Diabetic Retinopathy

Estimates done between 2005 and 2008 suggest that 28.5% of diabetics over the age of 40 in the United States had diabetic retinopathy and vision-threatening problems [42]. Low-grade chronic inflammation has been implicated in the pathogenesis of diabetic retinopathy [43]. The retina of diabetic individuals has a particular lipid profile [44]. COX-2 increases in the retina of diabetic animals, which contributes to abnormal production of PG [45].

5-LO-derived 5-HETE is the major proinflammatory eicosanoid, being five times higher in the vitreous of diabetics versus nondiabetics patients [46]. Mice* null* for the 5-LO gene demonstrated a minor inflammatory reaction [47–49]. Mice deficient in 5-LO had significantly less degeneration of retinal capillaries induced by diabetes, less superoxide generation, and less nuclear factor (NF)-kB expression [50]. Therefore, the generation of LTs could contribute to chronic inflammation and retinopathy in diabetes [51].

In addition, a hyperglycemic environment causes the release of 5-LO metabolites, LTA_4_ and LTB_4_. Retinas from both nondiabetic and diabetic mice are unable to produce LT or 5-LO mRNA. However, it was demonstrated that transcellular delivery of LTA_4_, from bone marrow-derived cells to retinal cells, results in the generation of LTB_4_/LTC_4_ [52]. LTC_4_ induces vascular permeability after binding with the retinal microvascular endothelial cells, and LTB_4_ coordinates proinflammatory pathways and superoxide generation, which may contribute to endothelial cell death and capillary degeneration, in turn contributing to chronic inflammation and diabetic retinopathy development [53].

### 2.3. Diabetic Peripheral Neuropathy

Estimates suggest 50% of diabetic patients have diabetic peripheral neuropathy, which affects the sensorimotor and autonomic parts of the peripheral nervous system [54–56]. Few studies describe the involvement of the eicosanoid pathway in DPN. In streptozotocin-induced rats, the intrathecal administration of COX-2 inhibitors, but not of COX-1 or COX-3 inhibitors, had an antihyperalgesic effect, supporting the importance of spinal COX-2 in DPN [57]. Pain may be attributed to the action of PGE_2_ on peripheral sensory neurons and on central sites within the spinal cord and the brain [58].

### 2.4. Diabetic Cardiovascular System

Impaired endothelial function is described in diabetes [59–61]. COX-2 expression and dilator prostaglandin synthesis increase in the coronary arterioles of diabetic patients [62]. Venous smooth muscle cells express more COX-2 and release more PGE_2_ when stimulated by a mix of inflammatory cytokines [63]. PGE_2_ causes pyrexia, hyperalgesia, and arterial dilation [58, 64]. PGE_2_ may act as a mediator of active inflammation, promoting first local vasodilatation, then the recruitment and activation of neutrophils, macrophages, and mast cells [65–68]. Deregulation of PGE_2_ synthesis leads to a wide range of pathological conditions [69]. In a normal cardiovascular system, PG_12_ acts as a potent vasodilator and TXA_2_ as a vasoconstrictor [70, 71]. The presence of both PGI_2_ and TXA_2_ maintains the normal physiology of the circulatory system [72]. In addition, the myocardium of diabetic and healthy rats does not differ in PG_12_ and PGE_2_ [73].

CYP-450-derived eicosanoids 12-HETE and 20-HETE, along with other inflammatory components in diabetic patients, lower the activity of endothelial progenitor cell function. Diabetic vascular complications are associated with reduced vascular regenerative potential and nonfunctional endothelial progenitor cell [79].

In sum, imbalanced levels of eicosanoids can induce modification of the microenvironment in the kidneys, eyes, nerves, and cardiovascular system and contribute to the progression of diabetes pathogenesis. Eicosanoid compounds have been studied as targets for drug development to control diabetes progression (Table 1). Thus, we reviewed drugs based on lipid mediators that are involved in diabetes complications.

## 3. Lipid Mediators in Modulation of Diabetes Complications

When celecoxib, a COX-2 inhibitor, was administered as therapy for diabetic nephropathy in a type 1 diabetes (T1DM) population, COX-2-dependent factors neutralized the angiotensin II effect in the renal microcirculation; further, this effect was greater in women with uncomplicated T1DM than in men [74]. These gender differences could be explained by higher plasma prostanoid found in female animals, an effect that may be estrogen mediated [80–83].

Lower modified levels of PGE_2_ relate to changes in the kidney microenvironment and the progression of diabetic nephropathy; thus, PGE_2_ and its action are also important targets for drug development [84]. The PGE_2_-EP4 pathway contributes to the progression of tubule interstitial fibrosis, and the chronic administration of EP4-agonist in mice, exacerbated inflammation via IL-6, and consequently albuminuria and fibrosis [85]. Additionally, EP4-agonist mediates hyperfiltration in the glomerulus in the early stages of diabetes [86, 87]. Diabetes inflammatory state and chemokine production also increased when mice (T1DM model) were treated with an EP4 agonist [85] and upregulated the development of immune responses Th1 and Th17 [88]. On the other hand, EP receptor antagonists inhibited Th1 and Th17 response [89, 90]. In summary, the activation of the EP4 receptor exacerbates albuminuria levels, inflammation, and fibrosis. COX-2 inhibition reduces albuminuria in renal disease in rats [91]. Recently, using PGE_1_ in diabetic nephropathy patients in different disease stages decreased proteinuria and albuminuria [92].

Treating diabetic rats with 50 mg/Kg of aspirin plus 2 mg/Kg of meloxicam (a COX-2 inhibitor) reduced leukocyte adhesion and suppression of the blood-retinal barrier breakdown. This combined dose also reduced retinal ICAM-1 expression, and aspirin alone reduced the expression of C11a, CD11b, and CD18. Together, aspirin and meloxicam reduced the level of TNF-*α* [93]. Among diabetic patients, 330 mg of aspirin significantly slowed the development of retinal microaneurysms in diabetic retinopathy [75]. Another controlled trial showed that celecoxib reduced vascular leakage in diabetic patients with diabetic retinopathy [76].

Topical administration of nonsteroidal anti-inflammatory drugs (NSAIDs) compared to nontopical administration minimizes systemic exposure to the drug, such that topical NSAIDs can help enhance intraocular penetration. Diabetic patients exhibited elevated plasma IL-8 and elevated vitreous PGE_2_ and IL-8 [78, 94]. Exposure to PGE_2_ induces IL-8 gene transcription in human T cells [95]. The binding of IL-1*β*, TNF-*α*, and IFN-*γ* also stimulates human retinal pigment epithelial cells to express IL-8 [96]. One study provides direct clinical evidence that topical ocular ketorolac tromethamine (0.45% NSAID) reduces vitreous IL-8 in patients with proliferative diabetic retinopathy [97].

One study found that latanoprost (a PGF_2*α*_ agonist) used topically significantly reduced dilation of retinal arterioles in type I diabetes patients with diabetic retinopathy, whereas topical diclofenac had no significant effect [77]. In diabetic rats, celecoxib lowered the synthesis of PGE_2_ in the retina (a result attributed to selective COX-2 inhibition, since COX-1 inhibitor did not have this effect) [98]. In addition, another COX inhibitor, nepafenac, inhibits increased retinal PG production and leukocyte adhesion in the retinal vessels of diabetes-induced rats [51].

In peripheral arterial diseases, the goal of treatment is to improve symptoms and prevent cardiovascular events [99]. Beraprost sodium is an analogue active PG_12_ with antiplatelet and vasodilating properties [100, 101]. Oral administration of beraprost sodium to diabetic patients improved sensations described as burning/hot, electric, sharp, achy, and tingling [100]. Beraprost improves symptoms by dilating peripheral vessels and increasing blood flow to the skin [102], and it can also improve painful peripheral neuropathy over a period of 8 weeks [103].

## 4. Future Perspectives on Eicosanoids

Components of the eicosanoid pathway have a fundamental role in the development of inflammation. As seen in this review, several studies have established that they participate in the progression of diabetes and its complications. Eicosanoids may act as pro- or anti-inflammatory. Currently, PG agonist and COX-1 and/or COX-2 inhibitors are the most promising tools to control diabetes complications, showing good results and promise for the future. Future studies should aim to unveil the function of specific receptors and enzymes acting in more specific targets available only in certain organs, such as the kidneys, eyes, vessels, or nerves.

## Figures and Tables

**Table 1:** Eicosanoid compounds as targets for drug development to control diabetes progression.

Drug	Target	Condition	Consideration	Reference
Celecoxibe	COX-2 inhibitor	Diabetes nephropathy	Female patients received higher dose of PGs vasodilator to maintain blood vessel function than male patients.	[74]
Aspirin	Nonselective COX inhibitor	Diabetes retinopathy	Delay in development of retinal microaneurysms in DR.	[75]
Celecoxibe	COX-2 inhibitor	Diabetes retinopathy	Reduction of vascular leakage.	[76]
Latanoprost	PGF2*α* agonist	Diabetes retinopathy	Reduces the diameter of dilated retinal arterioles.	[77]
Ketorolac tromethamine	Nonselective COX inhibitor	Diabetes retinopathy	Patients with suspected or visible fibrovascular proliferation demonstrated a reduction in IL-8 and platelet-derived growth factor levels in vitreous humor.	[78]
